# Giant magnetic splitting inducing near-unity valley polarization in van der Waals heterostructures

**DOI:** 10.1038/s41467-017-01748-1

**Published:** 2017-11-16

**Authors:** Philipp Nagler, Mariana V. Ballottin, Anatolie A. Mitioglu, Fabian Mooshammer, Nicola Paradiso, Christoph Strunk, Rupert Huber, Alexey Chernikov, Peter C. M. Christianen, Christian Schüller, Tobias Korn

**Affiliations:** 1 Institut für Experimentelle und Angewandte Physik, Universität Regensburg, D-93040 Regensburg, Germany; 2 High Field Magnet Laboratory (HFML–EMFL), Radboud University, 6525 ED Nijmegen, The Netherlands

## Abstract

Monolayers of semiconducting transition metal dichalcogenides exhibit intriguing fundamental physics of strongly coupled spin and valley degrees of freedom for charge carriers. While the possibility of exploiting these properties for information processing stimulated concerted research activities towards the concept of valleytronics, maintaining control over spin–valley polarization proved challenging in individual monolayers. A promising alternative route explores type II band alignment in artificial van der Waals heterostructures. The resulting formation of interlayer excitons combines the advantages of long carrier lifetimes and spin–valley locking. Here, we demonstrate artificial design of a two-dimensional heterostructure enabling intervalley transitions that are not accessible in monolayer systems. The resulting giant effective *g* factor of −15 for interlayer excitons induces near-unity valley polarization via valley-selective energetic splitting in high magnetic fields, even after nonselective excitation. Our results highlight the potential to deterministically engineer novel valley properties in van der Waals heterostructures using crystallographic alignment.

## Abstract (web summary)

In transition metal dichalcogenide monolayers, the spin and valley degrees of freedom are strongly coupled. Here, the authors engineer a WSe_2_/MoSe_2_ heterostructure in which inter-valley transitions of interlayer excitons exhibit a giant splitting and near-unity polarization in a magnetic field.

## Introduction

The materials combined in the studied heterostructure are monolayers of transition metal dichalcogenides (TMDCs) where MX_2_ denotes M = Mo, W and X = S, Se, Te. These systems were shown to host direct optical transitions in the visible spectral range at two inequivalent valleys in momentum space, labeled K+ and K−, which are situated at the corners of the hexagonal Brillouin zone^1–3^. Since spin and valley index of charge carriers are coupled at the K points due to the broken inversion symmetry in the monolayer combined with strong spin–orbit coupling, it is possible to selectively address and read out the valley index optically by helicity-resolved measurements^4–7^. However, as the two valleys are linked by time-reversal symmetry, external fields are required to break their energy degeneracy, an issue of central importance for future valleytronic devices. Recently, the effective manipulation of the valley pseudospin energy has been demonstrated for monolayer TMDCs by magnetic^8–15^ and electric fields^16,17^. However, the extremely short lifetimes of excitons^18,19^ and the fast polarization dephasing mechanisms^20^ render the implementation of individual TMDC monolayers challenging for valleytronics.

At the same time, the rapid development of transfer techniques has opened up a vast parameter space of artificial van der Waals heterostructures, where different two-dimensional (2D) materials are deterministically stacked upon each other^21^. For TMDCs, the resulting type II band alignment and subsequent rapid charge transfer^22^ leads to the formation of interlayer excitons (IEXs), where electrons and holes are situated in different layers^23–28^. For stacking angles close to 0° (AA stacking) and 60° (AB stacking), negligible momentum mismatch allows radiative recombination of charge carriers at the K points^29,30^, leading to pronounced light emission from IEXs below the energies of the individual monolayer transitions. Nevertheless, due to the spatial separation, the wavefunction overlap in the out-of-plane direction is reduced, facilitating the potential for long lifetimes of the interlayer excitons in 2D heterostructures^27^, further supplemented by the possibility of spin–valley injection in close analogy to the monolayer systems^28^.

Here, we show that the specific alignment of atomically thin layers in a heterostructure leads to intriguing fundamental physics, exclusive for such artificial systems and allowing for highly efficient external manipulation of the spin–valley degrees of freedom. In particular, we demonstrate that the magnetic coupling of electronic transitions can be strongly enhanced in AB-stacked TMDC heterobilayers, exhibiting a giant magnetic valley splitting with an effective *g* factor (*g*_eff_) of about −15 that exceeds typical values for both TMDC monolayers and more conventional nonmagnetic semiconductor heterostructures by far. Of central consequence is the resulting field-induced valley polarization of the long-lived charge carriers, even though both valleys in the two constituent materials are initially equally populated. The degree of polarization reaching near-unity values arises entirely due to the strong degeneracy lifting under magnetic fields. It emerges from the specific arrangement of the individual layers of our heterostructure in momentum space, which is not attainable in TMDC monolayer systems, enabling momentum-allowed optical transitions between valleys of different index. This leads to a situation where the sum of conduction- and valence-band valley magnetic moments contributes to significantly enhance the valley splitting in an external magnetic field compared to a monolayer system.

## Results

### Characterization of the WSe_2_/MoSe_2_ heterostructure

**Fig. 1:**
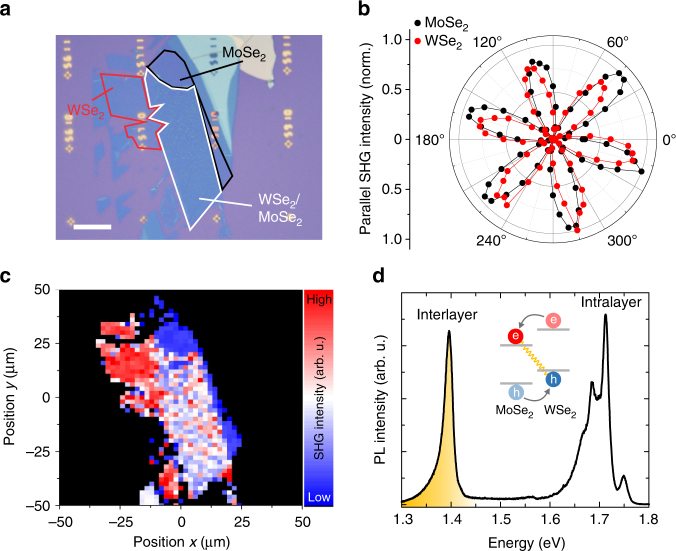
Interlayer excitons in a WSe_2_/MoSe_2_ heterostructure with nearly 60° angle alignment. **a** Optical micrograph of the WSe_2_/MoSe_2_ heterostructure under study. The white framed area depicts the region where the two materials overlap vertically. The scale bar is 25μm. **b** Angle-dependent plot of the parallel component of the SHG intensity of the individual monolayers, indicating the armchair directions of the monolayers. The relative angle between the monolayers amounts to about 54°. **c** Spatial scan of the sample where the total SHG intensity is recorded for each datapoint. The region of the heterostructure shows clear destructive interference of the SHG signal with respect to the individual layers. **d** PL spectrum taken on the heterostructure at 4 K. The emission stemming from interlayer excitons is spectrally well separated from the intralayer luminescence. The inset schematically depicts the type II band alignment of the heterostructure which leads to a spatial separation of electrons and holes

The heterostructure under study (shown in Fig. 1a) consists of a monolayer of WSe_2_ transferred on top of a MoSe_2_ monolayer, exfoliated onto a SiO_2_/Si substrate. During the transfer process, the well-cleaved axes of the monolayers are deterministically aligned parallel to each other, ensuring a twist angle of either nearly 0° or 60°. The relative angle of the stacking configuration is confirmed through spatially resolved second-harmonic generation (SHG) spectroscopy^31^. Figure 1b illustrates the intensity of the parallel component of the SHG signal of the two individual monolayer materials in a polar plot. By fitting the data with a cos^2^(3*θ*) function we directly obtain a relative stacking angle *θ* of either 6 ± 1° or 54 ± 1° (the two possibilities stem from the phase insensitivity of the SHG intensity measurement on a single layer). To clarify the precise alignment, we perform a spatial scan of the resulting heterostructure where the total SHG intensity is recorded at each position, as shown in Fig. 1c. In the overlapping region of the two layers denoted by the white framed area in Fig. 1a, we clearly observe a pronounced destructive interference of the SHG signal with respect to the individual monolayers, consistent with a nearly 60° stacking configuration^31^. Thus, we conclude that the sample has an AB-like stacking configuration with a relative angle of 54 ± 1°.

A characteristic photoluminescence (PL) spectrum of the heterostructure recorded at 4 K is shown in Fig. 1d. In line with recent reports, it consists of two separate spectral regions:^27,28^ The intralayer transitions between 1.6 and 1.8 eV result from intralayer excitonic recombination in the constituent monolayers (WSe_2_ and MoSe_2_). At the same time, as depicted in the inset of Fig. 1d, rapid charge transfer leads to the formation of interlayer excitons at around 1.4 eV, significantly lower in energy than the intralayer transitions (Additional characterization measurements are presented in Supplementary Note 1).

### Magneto-PL measurements of the interlayer exciton

**Fig. 2:**
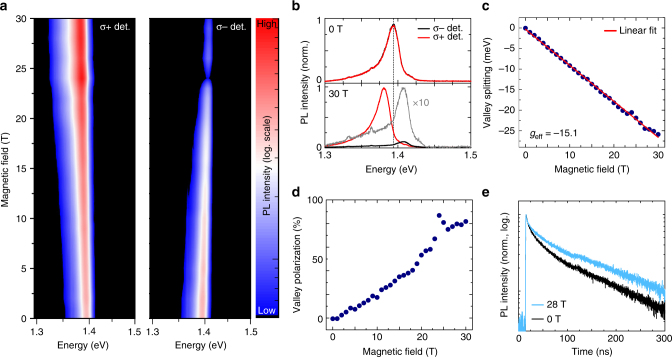
Magnetic field dependence of interlayer excitons. **a** False color representation of the interlayer exciton PL for *σ*+ and *σ*− polarized detection as a function of out-of-plane magnetic field up to 30 T. The excitation is performed with linearly polarized light. For better clarity the PL intensity is plotted on a logarithmic scale. **b** Comparison of PL spectra of the interlayer exciton for 0 T and 30 T. At 0 T, both polarizations show the same energy and intensity. At 30 T, the energy degeneracy is fully lifted and the emission stems almost exclusively from the *σ*+ transition. **c** Corresponding valley-selective splitting of the interlayer exciton. The solid line corresponds to a linear fit of the data, yielding an effective *g* factor of −15.1 ± 0.1. **d** Magnetic-field-induced valley polarization of the interlayer exciton. **e** Time-resolved PL of the interlayer exciton for *B* = 0 T and *B* = 28 T

We now turn to the valley-resolved measurements of the interlayer transitions in an out-of-plane magnetic field (Faraday geometry). The optical experiments are carried out by exciting the sample with linearly polarized light (laser energy 1.94 eV), initially populating both valleys in the two monolayer constituents of the heterostructure equally. The emission is subsequently analyzed in a circularly polarized basis, allowing us to directly access the resulting valley-selective splitting of the transitions and quantify the degree of polarization. Figure 2a shows the spectral evolution of the interlayer excitons for both detection polarizations in magnetic fields up to 30 T. Two main observations are immediately apparent from the data. First, the peak energy degeneracy of the interlayer transitions is lifted for fields *B* > 0. For rising magnetic fields, the energy of the *σ*+ polarized component decreases monotonically while it increases for the *σ*− polarized component. Second, the intensity of the interlayer exciton strongly depends on the detected polarization in the magnetic field. The *σ*+ and *σ*− polarized components exhibit a drastic increase and decrease in intensity, respectively, as the magnetic field is increased. These observations are further illustrated in Fig. 2b, where the two polarization configurations for *B* = 0 T and *B* = 30 T are directly compared. While at *B* = 0 T the two circularly polarized emission peaks are of same energy and intensity, the energy splitting between the two valley configurations amounts to about 26 meV for *B* = 30 T, exceeding the linewidth of the two transitions. Also, the luminescence stems almost exclusively from the *σ*+ transition, with the *σ*− emission being strongly suppressed.

For the quantitative analysis of the data, we use Gaussian fit functions, and extract the PL peak positions of the interlayer transition for both polarizations as function of the magnetic field. The resulting valley splitting, presented in Fig. 2c, clearly follows a linear dependence. Using the definition of the splitting as Δ*E* ^IEX^ = *E* ^*σ*+^−*E* ^*σ*−^ = *g*_eff_ *μ*_*B*_ *B*, where *μ*_*B*_ is the Bohr magneton (≈58 μeV/T), we extract *g*_eff_ = −15.1 ± 0.1 for the interlayer exciton.

The corresponding degree of the valley polarization, defined as $P = \left( {I_{\sigma ^ + } - I_{\sigma ^ - }} \right)/\left( {I_{\sigma ^ + } + I_{\sigma ^ - }} \right)$, is presented in Fig. 2d. While it is strictly zero at *B* = 0, as expected, the emission is strongly polarized under the external magnetic field, exceeding values of 80% for the highest fields up to 30 T (see Supplementary Note 2 for a discussion of the enhanced valley polarization in the data around *B* = 24 T). We further note that such overall high degree of field-induced polarization is particularly remarkable given the fact that both valleys are initially equally populated in the experiment. Using time-resolved PL measurements (see Methods) we also track the decay dynamics of the interlayer exciton in the magnetic field. The sample is excited linearly and the total PL intensity is detected. The data are presented in Fig. 2e. The decay dynamics exhibit a complex nonexponential decay, with a 1/*e* time constant of about 40 ns at 0 T followed by a longer decay (>100 ns) at later times and exceeding typical values for individual TMDC monolayers by several orders of magnitude. The lifetime further increases with rising magnetic field, with the 1/*e* constant reaching 70 ns at 28 T and the longer component increasing beyond 200 ns (see Supplementary Note 3 for more detailed data). Applying an external magnetic field therefore allows us to not only to generate strongly valley-polarized carriers, but also maintain the interlayer emission on very long timescales.

The determined value of about −15 for the effective *g* factor of the interlayer exciton is in stark contrast to experimentally determined effective *g* factors of excitons in individual TMDCs, found to be around −4 in most cases^9–12,14,15^. As we show in the following, this anomalously high effective *g* factor results from spin-allowed intervalley transitions in our AB-stacked heterostructure, which enables us to access novel valley physics in an external magnetic field. In monolayers, the magnitude of the magnetic coupling is often understood in terms of a simplified semiquantitative model, including three contributions, namely the spin, the atomic orbitals, and the valley magnetic moment^8–12^. Since the optical transitions are spin conserving between conduction and valence band, the net contribution from the spin to the energy splitting of the respective resonances is zero. On the other hand, only the valence bands carry a non-zero magnetic moment *μ*_*l*_ from the atomic orbitals with *μ*_*l*_ = 2 for the K+ valley and *μ*_*l *_= −2 for the K− valley, leading to an overall splitting between the valley-selective transitions of −4*μ*_*B*_ *B*. The third contribution, the valley magnetic moment *μ*_*k*_, arises from the self-rotation of the Bloch wavepackets^32^. It is defined by $\pm \mu _k^c = m_0/m_e$ for the conduction band and $\pm \mu _k^v = m_0/m_h$ for the valence band in the K+/K− valley, respectively. As the optically allowed transitions in a monolayer take place between valleys of the same index (intravalley), these contributions cancel out almost entirely. For intervalley transitions in a monolayer system, the contributions from the valley magnetic moments add up, leading to higher *g* factors^33^. These transitions cannot be optically accessed in a pristine monolayer system due to their high momentum mismatch. However, they may be responsible for the large *g* factors observed in defect-related emission^34–38^. Therefore, the total field-induced magnetic shift for a monolayer TMDC can be written as Δ*E* ^1L^ = *E* ^*σ*+^−*E* ^*σ*−^ = −(4−2(*m*_0_/*m*_*e*_−*m*_0_/*m*_*h*_))*μ*_*B*_ *B *≈ −4*μ*_*B*_ *B*, as *m*_*h*_ ≈ *m*_*e*_ in many cases. The deviations from this value are attributed both to nonequivalent effective masses of electrons and holes and the complexities of the orbital contributions to the energetic shift beyond the simplified model^12^.

**Fig. 3:**
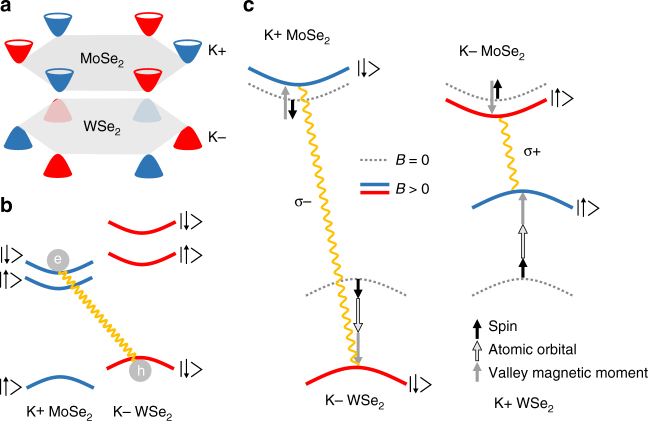
Giant magnetic valley splitting in an AB-stacking configuration. **a** Momentum space arrangement of the relevant band extrema in an AB-stacked WSe_2_/MoSe_2_ heterostructure. Blue (red) depicts electronic bands from the K+ (K−) valleys. **b** Type II band alignment of MoSe_2_ and WSe_2_ for the AB-stacking configuration, indicating spin-allowed optically bright interlayer transitions. Arrows indicate spin-up and spin-down states. **c** Evolution of the transitions such as indicated in **b** with positive applied magnetic field. Dashed lines indicate the situation for *B* = 0. The arrows depict the possible contributions to the valley-selective splitting (black for spin, black framed for atomic orbital and gray for valley magnetic moment)

In an AB-stacked heterostructure, however, we encounter a markedly different situation for optically bright transitions. Figure 3a schematically depicts the configuration of the Brillouin zones for interlayer excitons in an AB-stacked WSe_2_/MoSe_2_ heterostructure. Here, the optical transitions take place between the K− valley of WSe_2_ and the K+ valley of MoSe_2_ (and by symmetry, also from K+ in WSe_2_ to K− in MoSe_2_). Hence, in contrast to monolayer systems, the optical transitions are not valley conserving for AB-stacked heterobilayers. This is further illustrated in Fig. 3b which shows one of the two interlayer transitions. After the optical excitation and following fast charge transfer, electrons in the upper conduction band of MoSe_2_ reside in the K+ valley whereas the holes in WSe_2_ are located in the K− valley (here, we use the convention where the vacant electron states and the corresponding holes in the valence band are defined to have the same momenta). This configuration, in analogy to tungsten-based monolayer TMDCs, leads to the spin-allowed and optically bright transitions involving the upper conduction band of MoSe_2_ while a transition from the lower conduction band is not spin conserving and thus optically dark. The anomalous situation in momentum space directly impacts the valley splitting of the interlayer exciton in an external magnetic field, which is schematically depicted in Fig. 3c.

In analogy to the monolayer system, the contribution to the valley splitting from spin cancels out and the magnetic moment from the atomic orbitals *μ*_*l*_ in the valence bands results in an expected energetic splitting of −4*μ*_*B*_ *B*. The contributions from the valley magnetic moments *μ*_*k*_, however, now have the opposite sign for the conduction and valence bands and thus add up instead of canceling out as it is the case for individual monolayers. This signifies the main magnetic property of the interlayer transitions in AB-stacked heterostructures with the energetic shifts of the valley magnetic moments evolving antiparallel in the conduction and valence bands in a magnetic field. For the *σ*− transition in Fig. 3c this leads to a drastic increase of the transition energy when a magnetic field is applied. For the same reasons, a strong decrease occurs for the *σ*+ transition, since the two valley configurations are linked by time-reversal symmetry. The total valley-selective splitting of the interlayer transition Δ*E* ^IEX^ induced by the magnetic field then amounts to Δ*E* ^IEX^ = *E* ^*σ*+^−*E* ^*σ*−^ = −(4 + 2(*m*_0_/*m*_*e*_ + *m*_0_/*m*_*h*_))*μ*_*B*_ *B*. Using calculated values^39^ for the effective masses of electrons (*m*_*e*_ = 0.57*m*_0_) in the upper conduction band of MoSe_2_ and holes (*m*_*h*_ = 0.36*m*_0_) in the valence band of WSe_2_ we obtain *g*_eff_ = −13.1 for the interlayer transition, in close agreement with the experimentally determined value of *g*_eff_ = −15.1 ± 0.1.

## Discussion

The presence of large band offsets in TMDC heterostructures combined with the angle alignment of the nonequivalent valleys thus yields interlayer transitions with an unusually large total effective *g* factor, which is a powerful lever to exploit the valley degree of freedom. Even higher effective *g* factors can be expected when suitable van der Waals materials with lower effective masses are combined. Thus, even for nonselective injection with respect to spin–valley degrees of freedom, strong spin–valley polarization in an applied magnetic field is expected already at thermal equilibrium steady-state conditions, as demonstrated in our experiment. This finding is of particular importance when-above bandgap optical excitation or electrical injection are considered, both being most common scenarios in devices. Additionally, we envision the use of local (stray or exchange) magnetic fields generated by microstructured ferromagnets to define valley-selective potential landscapes for interlayer excitons. These local, valley-selective potentials may be used to filter or trap excitons with a specific valley polarization, and the large energy splitting resulting from the effective *g* factor will enable operation of such devices well above liquid-helium temperatures. Finally, the demonstrated stability of the spin–valley polarization through the complete lifting of valley degeneracy in artificial heterostructures provides a highly promising route towards the implementation of the spin–valley degree of freedom for future applications in the fields of quantum computation and nanophotonics.

## Methods

### Sample fabrication

The WSe_2_/MoSe_2_ heterostructure was fabricated by means of an all-dry transfer procedure^40^ on a Si/SiO_2_ substrate. The constituent monolayer samples were obtained by mechanical exfoliation of bulk crystals (HQGraphene). After the transfer process, the sample was annealed for 5 h at 150 °C in high vacuum.

### Second-harmonic generation spectroscopy

SHG measurements were carried out at room temperature with a Ti:sapphire laser (pulse length 100 fs, central wavelength 810 nm) focused on the sample via a 40 × microscope objective. The signal was coupled into a grating spectrometer and detected with a CCD camera. For polarization-dependent measurements, the laser light was linearly polarized and the reflected light was analyzed by the same polarizer, thereby selecting the parallel signal component of the SHG. The sample was rotated by a mechanical stage in order to obtain angle resolution. For mapping of the total SHG intensity, the sample was excited using circularly polarized light without any polarization analysis in the detection. The sample was scanned under the microscope using a motorized x–y stage and the total SHG intensity was recorded for each sample position.

### Magneto-PL spectroscopy

The sample was placed on a x–y–z piezoelectric stage and cooled down to 4.2 K in a cryostat filled with liquid helium. Magnetic fields up to 30 T were applied by means of a resistive magnet in Faraday configuration. For static PL measurements, laser light at an energy of 1.94 eV was focused onto the sample with a microscope objective resulting in a spot size of ~4μm. The polarization of the PL was analyzed with a quarter-wave plate and a linear polarizer. Using a nonpolarizing beam splitter, the backscattered PL was guided to the spectrometer and detected with a liquid nitrogen-cooled CCD. Time-resolved PL measurements were carried out with a pulsed diode laser (laser energy 1.80 eV, repetition rate 2.5 Mhz) which was synchronized to an avalanche photodiode. The PL from the interlayer exciton was spectrally selected with a longpass filter.

### Data availability

The data that support the findings of this study are available from the corresponding authors upon request.

## Electronic supplementary material

Supplementary Information

Peer Review File

## References

[1] Mak KF, Lee C, Hone J, Shan J, Heinz TF (2010). Atomically thin MoS_2_: a new direct-gap gemiconductor. Phys. Rev. Lett..

[2] Splendiani A (2010). Emerging photoluminescence in monolayer MoS_2_. Nano Lett..

[3] Xu X, Yao W, Xiao D, Heinz TF (2014). Spin and pseudospins in layered transition metal dichalcogenides. Nat. Phys..

[4] Xiao D, Liu GB, Feng W, Xu X, Yao W (2012). Coupled spin and valley physics in monolayers of MoS_2_ and other group-VI dichalcogenides. Phys. Rev. Lett..

[5] Mak KF, He K, Shan J, Heinz TF (2012). Control of valley polarization in monolayer MoS_2_ by optical helicity. Nat. Nanotech..

[6] Zeng H, Dai J, Yao W, Xiao D, Cui X (2012). Valley polarization in MoS_2_ monolayers by optical pumping. Nat. Nanotech..

[7] Schaibley JR (2016). Valleytronics in 2D materials. Nat. Rev. Mater..

[8] Aivazian G (2015). Magnetic control of valley pseudospin in monolayer WSe_2_. Nat. Phys..

[9] Li Y (2014). Valley splitting and polarization by the Zeeman effect in monolayer MoSe_2_. Phys. Rev. Lett..

[10] Srivastava A (2015). Valley Zeeman effect in elementary optical excitations of monolayer WSe_2_. Nat. Phys..

[11] MacNeill D (2015). Breaking of valley degeneracy by magnetic field in monolayer MoSe_2_. Phys. Rev. Lett..

[12] Wang G (2015). Magneto-optics in transition metal diselenide monolayers. 2D Mater..

[13] Mitioglu AA (2015). Optical investigation of monolayer and bulk tungsten diselenide (WSe_2_) in high magnetic fields. Nano Lett..

[14] Stier AV, McCreary KM, Jonker BT, Kono J, Crooker SA (2016). Exciton diamagnetic shifts and valley Zeeman effects in monolayer WS_2_ and MoS_2_ to 65 Tesla. Nat. Commun..

[15] Plechinger G (2016). Excitonic valley effects in monolayer WS_2_ under high magnetic fields. Nano Lett..

[16] Sie EJ (2014). Valley-selective optical Stark effect in monolayer WS_2_. Nat. Mater..

[17] Kim J (2014). Ultrafast generation of pseudo-magnetic field for valley excitons in WSe_2_ monolayers. Science.

[18] Poellmann C (2015). Resonant internal quantum transitions and femtosecond radiative decay of excitons in monolayer WSe_2_. Nat. Mater..

[19] Robert C (2016). Exciton radiative lifetime in transition metal dichalcogenide monolayers. Phys. Rev. B.

[20] Glazov MM (2014). Exciton fine structure and spin decoherence in monolayers of transition metal dichalcogenides. Phys. Rev. B.

[21] Geim AK, Grigorieva IV (2013). Van der Waals heterostructures. Nature.

[22] Hong X (2014). Ultrafast charge transfer in atomically thin MoS_2_/WS_2_ heterostructures. Nat. Nanotech..

[23] Kang J, Tongay S, Zhou J, Li J, Wu J (2013). Band offsets and heterostructures of two-dimensional semiconductors. Appl. Phys. Lett..

[24] Kosmider K, Fernández-Rossier J (2013). Electronic properties of the MoS_2_-WS_2_ heterojunction. Phys. Rev. B.

[25] Fang H (2014). Strong interlayer coupling in van der Waals heterostructures built from single-layer chalcogenides. Proc. Natl. Acad. Sci. USA.

[26] Lee CH (2014). Atomically thin p-n junctions with van der Waals heterointerfaces. Nat. Nanotech..

[27] Rivera P (2015). Observation of long-lived interlayer excitons in monolayer MoSe_2_-WSe_2_ heterostructures. Nat. Commun..

[28] Rivera P (2016). Valley-polarized exciton dynamics in a 2D semiconductor heterostructure. Science.

[29] Yu H, Wang Y, Tong Q, Xu X, Yao W (2015). Anomalous light cones and valley optical selection rules of interlayer excitons in twisted heterobilayers. Phys. Rev. Lett..

[30] Nayak PK (2017). Probing evolution of twist-angle-dependent interlayer excitons in MoSe_2_/WSe_2_ van der Waals heterostructures. ACS Nano.

[31] Hsu WT (2014). Second Harmonic generation from artificially stacked transition metal dichalcogenide twisted bilayers. ACS Nano.

[32] Xiao D, Yao W, Niu Q (2007). Valley-contrasting physics in graphene: Magnetic moment and topological transport. Phys. Rev. Lett..

[33] Koperski, M. et al. Optical properties of atomically thin transition metal dichalcogenides: observations and puzzles. *Nanophotonics* doi:10.1515/nanoph-2016-0165 (2017).

[34] Srivastava A (2015). Optically active quantum dots in monolayer WSe_2_. Nat. Nanotech..

[35] Koperski M (2015). Single photon emitters in exfoliated WSe_2_ structures. Nat. Nanotech..

[36] Chakraborty C, Kinnischtzke L, Goodfellow KM, Beams R, Vamivakas AN (2015). Voltage-controlled quantum light from an atomically thin semiconductor. Nat. Nanotech..

[37] He YM (2015). Single quantum emitters in monolayer semiconductors. Nat. Nanotech..

[38] Tonndorf P (2015). Single-photon emission from localized excitons in an atomically thin semiconductor. Optica.

[39] Kormányos A (2015). k. p theory for two-dimensional transition metal dichalcogenide semiconductors. 2D Mater..

[40] Castellanos-Gomez A (2014). Deterministic transfer of two-dimensional materials by all-dry viscoelastic stamping. 2D Mater..

