# Supplementary material for: Giant magnetic splitting inducing near-unity valley polarization in van der Waals heterostructures
Source: Nat Commun. 2017 Nov 16;8:1551. doi: 10.1038/s41467-017-01748-1 (PMC5691051; doi:10.1038/s41467-017-01748-1)
Supplement: Supplementary file 1 — Supplementary Information [file 41467_2017_1748_MOESM1_ESM.pdf]

Supplementary Note 1: Basic characterization of interlayer excitons in a $WSe_2/MoSe_2$ heterostructure

Supplementary Figure 1a shows the optical image of the $WSe_2/MoSe_2$-heterostructure under study where the two materials overlap in the white-framed area. A PL scan of the sample at 4 K with respect to the PL intensity in the spectral region of the interlayer exciton is shown in supplementary Figure 1b.

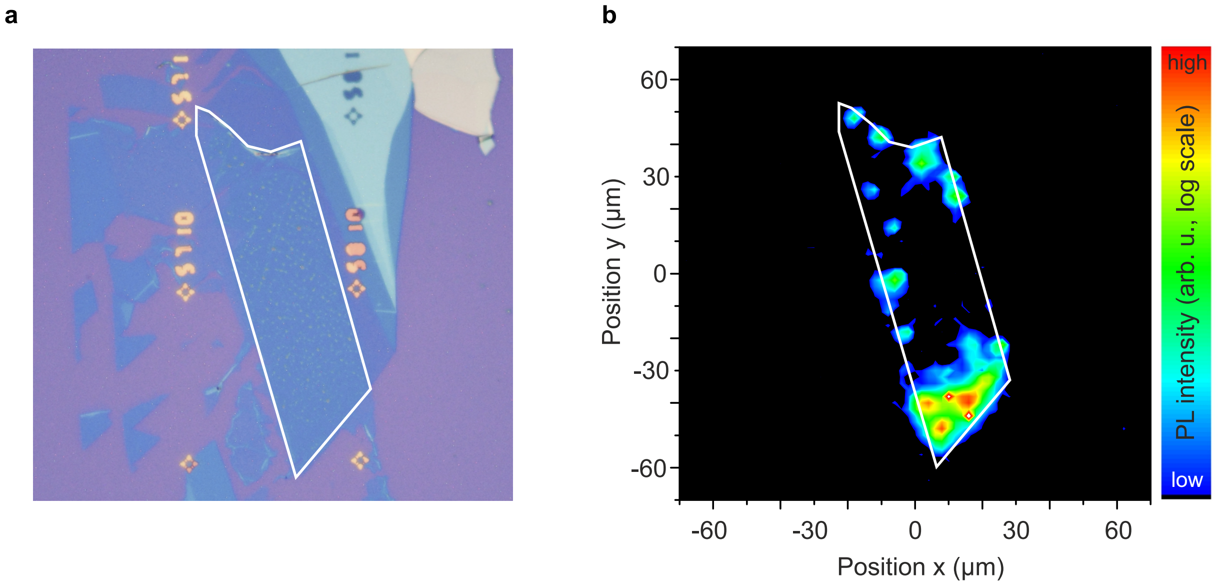

Supplementary Figure 1. PL scan of the sample at 4 K **a,** Optical micrograph of the $WSe_2/MoSe_2$ heterostructure under study. The white framed area depicts the region where the two materials overlap vertically. **b,** PL intensity with respect to the emission of the spectral region of the interlayer exciton.

The PL intensity of each point of the scan was obtained by an automated fitting routine using a single Gaussian. As can be seen, the emission from the interlayer exciton mostly stems from the lower part of the sample, covering an area of about 30 µm x 20 µm. Additionally several other smaller areas of the heterostructure also show emission from the interlayer exciton. It is worth noting that the presence of a large homogeneous area where the emission of the interlayer exciton occurs is in clear contrast to the situation of localized excitons which have been mainly observed at the edges and steps of monolayer samples. [1–5]. We presume, that in the remaining part of the sample, where no discernible emission from the interlayer exciton is present, the contact between the two materials is not sufficient.

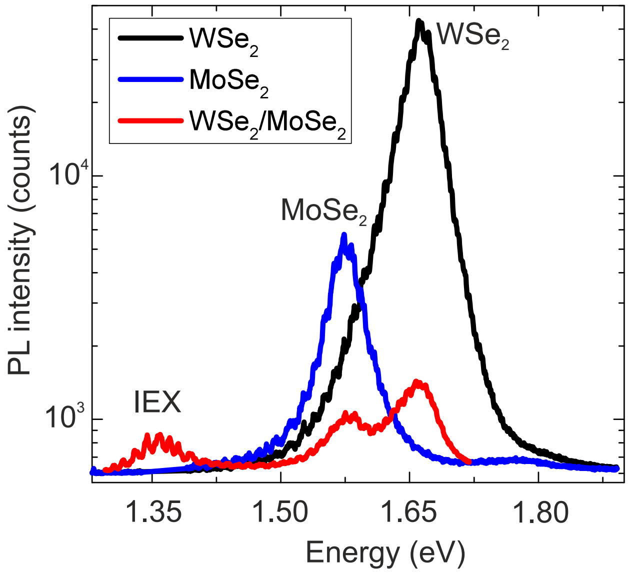

Supplementary Figure 2. Observation of interlayer excitons at room temperature. Comparison of room temperature spectra of the bare $MoSe_2$ and WSe_2 monolayers of the sample and a spectrum in the region where the two materials overlap.

Supplementary Figure 2 shows a comparison of the PL spectra for the different regions of the studied sample at room temperature. The emission from the individual monolayers is strongly quenched in the heterostructure, indicating ultra-fast charge transfer. More importantly, in the spectrum of the heterostructure we observe the emission from interlayer excitons at around 1.35 eV, in line with recent literature [6].

Supplementary Note 2: Anomalous behavior of the interlayer exciton at B=23-24 T

The behavior of the interlayer exciton with respect to intensity, energy and lifetime shows a slight anomaly at a magnetic field of around B=23-24 T as can be seen in Figures 2a,c,d of the main text. We would like to mention that this behavior of the interlayer exciton could be reproduced during various measurement cycles with different excitation conditions. At the same time, no anomalous deviation at B=23-24 T was present for other excitonic features in the same spectra, e.g. the A exciton of WSe_2, which is presented in Supplementary Figure 3. Here, both the valley splitting (Supplementary Figure 3a) and the deduced valley polarization (Supplementary Figure 3b) do not show an anomaly at around B=23-24 T. Therefore, we can mostly exclude external effects stemming from the overall setup which otherwise might be responsible for this feature. The obtained values for the effective g factor and the valley polarization of the WSe_2 A exciton are mostly in line with recent reports [7–10].

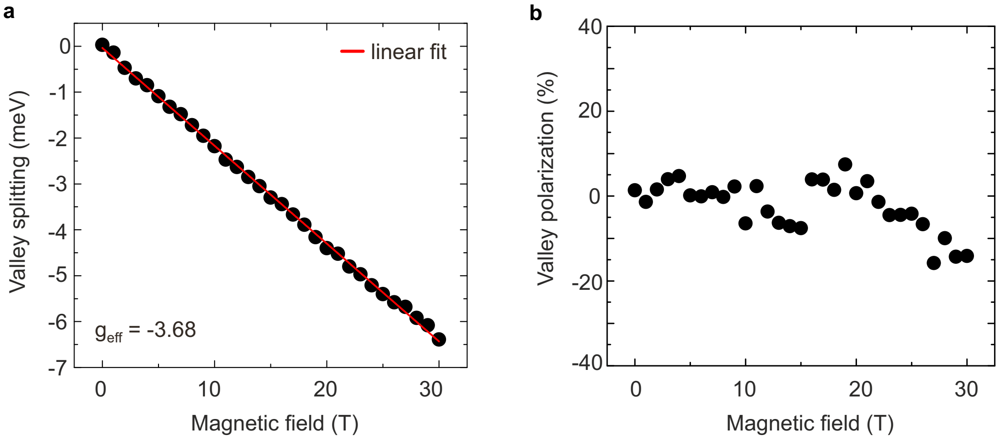

Supplementary Figure 3. Magnetic field dependence of the WSe_2 A exciton resonance in the heterostructure. **a,** Deduced valley splitting of the A exciton resonance of WSe_2. The solid red line corresponds to a linear fit of the data yielding an effective g factor of -3.68 ± 0.1. **b,** Magnetic-field-induced valley polarization of the WSe_2 A exciton.

A possible scenario for the interesting behavior of the interlayer exciton at around $B = 24$ T could be a level crossing of the conduction bands of $MoSe_2$ which might explain the unusual behavior of the valley polarization, effective g factor and lifetime of the interlayer exciton. In this context one can think of either a crossing of the bands within the same

valley or an inter-valley crossing where the lower conduction band of the K+ valley crosses the upper conduction band in the K− valley of $MoSe_2$. However, given the typical energy splitting of the conduction band of $MoSe_2$, we believe that both scenarios are unlikely.

Supplementary Figure 4 shows the expected evolution of the conduction bands of $MoSe_2$ which are linked by time reversal symmetry in an external out-of-plane magnetic field. Since the conduction bands mostly comprise of d-orbitals with m=0 we neglect the contribution from the atomic orbitals to the overall energy shift in both cases. A level-crossing of the conduction bands within a valley would take place if the energetic splitting of the conduction bands in the K+ or K− valley approaches a value near 0. For the conduction band in the K− valley of $MoSe_2$ this is not possible since the overall splitting will increase as can be seen in Figure 4. For the K+ valley, the splitting becomes in fact smaller with an applied magnetic field. In this case, the field-induced change of the bands is written as $\Delta E_{\text{intra}} = 2s_z\mu_B B + 2s_z\mu_B B$. For $B = 24\,\text{T}$ this yields a decrease of the splitting between the bands of about $\Delta E_{\text{intra}} \approx 2.8\,\text{meV}$.

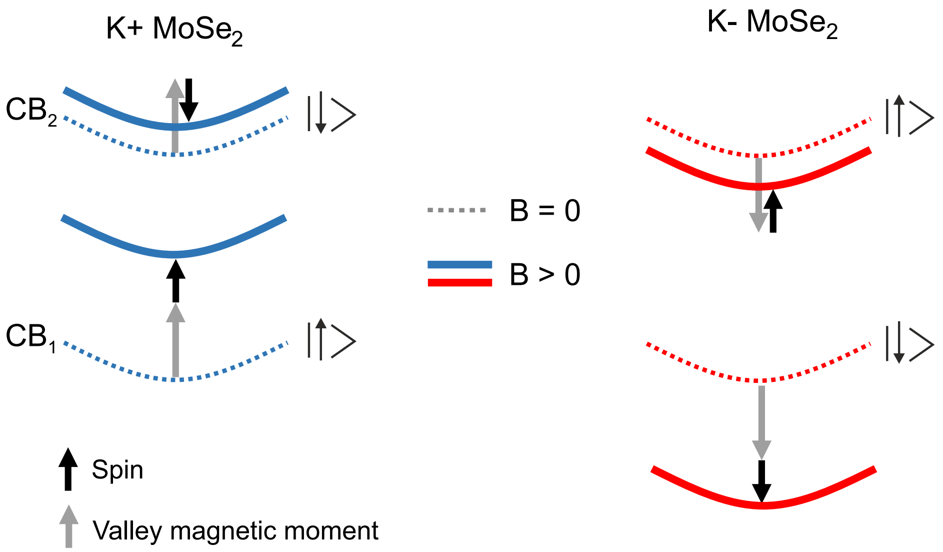

Supplementary Figure 4. Evolution of the conduction bands in $MoSe_2$ with applied magnetic field. Dashed lines indicate the energetic position of the spin-split conduction bands (CB_1 and CB_2) in $MoSe_2$ at $B = 0\,\text{T}$. The changes of the energetic levels by an external out-of-plane magnetic field are indicated by black arrows (spin) and grey arrows (valley magnetic moment).

For an inter-valley crossing between the lower conduction band in the K+ valley and the

upper conduction band in the K$-$ valley, we have to take into account only the energetic shifts from the valley magnetic moment, as the contributions from spin cancel out (see Supplementary Fig.4). The field-induced change is then written as $\Delta E_{\text{inter}} = m_0/m_e + m_0/m_e$, where m_0 and m_e are the mass of the free electron and the effective mass in the conduction band respectively. Using recently calculated values for the masses of the electron in $MoSe_2$ ($m_e = 0.57 m_0$) we obtain a shift of $\Delta E_{\text{inter}} \approx 4.9\,\text{meV}$.

Recent experimental data [11] and k·p calculations [12] however obtain a value for the conduction band splitting of $MoSe_2$ of around 20 - 30 meV which exceeds both the values for intra-valley splitting $\Delta E_{\text{intra}} \approx 2.8\,\text{meV}$ and inter-valley splitting $\Delta E_{\text{inter}} \approx 4.9\,\text{meV}$ at a magnetic field of 24 T by far. Thus, the applied magnetic fields are not sufficient in our case to lead to a level crossing in the conduction band.

Alternatively, the observed behavior may be related to resonantly enhanced intervalley phonon scattering when the conduction-band level splitting matches an optical phonon energy.

Supplementary Note 3: Time-resolved PL of interlayer excitons in an external magnetic field

Supplementary Figure 5a shows the $1/e$ constant of the PL decay of the interlayer exciton in the external magnetic field. The lifetime increases with rising magnetic field, with the $1/e$ constant reaching 70 ns at 28 T.

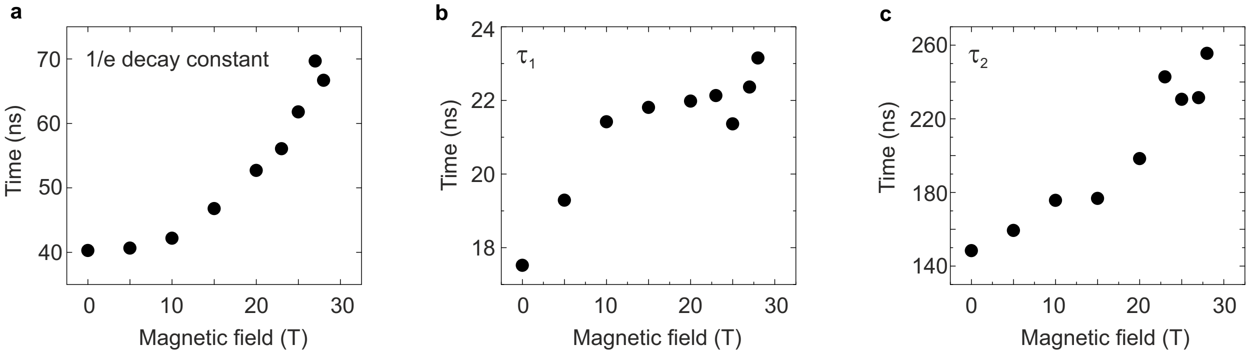

Supplementary Figure 5. Decay dynamics of interlayer excitons in a magnetic field. **a,** $1/e$ constant of the PL decay in dependence of external magnetic field. **b,c** Decay constants of the biexponential fit function τ_1 and τ_2 in dependence of external magnetic field.

The complex PL trace can also be fitted with a biexponential decay function. The obtained time constants τ_1 and τ_2 are shown in Supplementary Figures 5a and b respectively. As for the $1/e$ constant, both increase with rising magnetic field.

SUPPLEMENTARY REFERENCES

[1] Srivastava, A. *et al.* Optically active quantum dots in monolayer WSe_2. *Nat. Nanotech.* **10**, 491–496 (2015).

[2] Koperski, M. *et al.* Single photon emitters in exfoliated WSe_2 structures. *Nat. Nanotech.* **10**, 503–506 (2015).

[3] Chakraborty, C., Kinnischtzke, L., Goodfellow, K. M., Beams, R. & Vamivakas, A. N. Voltage-controlled quantum light from an atomically thin semiconductor. *Nat. Nanotech.* **10**, 507–511 (2015).

[4] He, Y.-M. *et al.* Single quantum emitters in monolayer semiconductors. *Nat. Nanotech.* **10**, 497–502 (2015).

[5] Tonndorf, P. *et al.* Single-photon emission from localized excitons in an atomically thin semiconductor. *Optica* **2**, 347 (2015).

[6] Rivera, P. *et al.* Observation of long-lived interlayer excitons in monolayer $MoSe_2$-WSe_2 heterostructures. *Nat. Commun.* **6**, 6242 (2015).

[7] Aivazian, G. *et al.* Magnetic control of valley pseudospin in monolayer WSe_2. *Nat. Phys.* **11**, 148–152 (2015).

[8] Srivastava, A. *et al.* Valley Zeeman effect in elementary optical excitations of monolayer WSe_2. *Nat. Phys.* **11**, 141–147 (2015).

[9] Wang, G. *et al.* Magneto-optics in transition metal diselenide monolayers. *2D Mater.* **2**, 034002 (2015).

[10] Mitioglu, A. A. *et al.* Optical Investigation of Monolayer and Bulk Tungsten Diselenide (WSe_2) in High Magnetic Fields. *Nano Lett.* **15**, 4387–4392 (2015).

[11] Wang, Z., Zhao, L., Mak, K. F. & Shan, J. Probing the Spin-Polarized Electronic Band Structure in Monolayer Transition Metal Dichalcogenides by Optical Spectroscopy. *Nano Lett.* **17**, 740–746 (2017).

[12] Kormányos, A. *et al.* $k \cdot p$ theory for two-dimensional transition metal dichalcogenide semiconductors. *2D Mater.* **2**, 022001 (2015).
