# Supplementary material for: Giant magnetic splitting inducing near-unity valley polarization in van der Waals heterostructures
Source: Nat Commun. 2017 Nov 16;8:1551. doi: 10.1038/s41467-017-01748-1 (PMC5691051; doi:10.1038/s41467-017-01748-1)
Supplement: Supplementary file 2 — Peer Review File [file 41467_2017_1748_MOESM2_ESM.pdf]

Editorial Note: this manuscript has been previously reviewed at another journal that is not operating a transparent peer review scheme. This document only contains reviewer comments and rebuttal letters for versions considered at Nature Communications.
